# Beyond genomics in Patescibacteria: A trove of unexplored biology packed into ultrasmall bacteria

**DOI:** 10.1073/pnas.2419369121

**Published:** 2024-12-12

**Authors:** Pooja Srinivas, S. Brook Peterson, Larry A. Gallagher, Yaxi Wang, Joseph D. Mougous

**Affiliations:** ^a^Department of Microbiology, University of Washington, Seattle, WA 98109; ^b^HHMI, University of Washington, Seattle, WA 98109; ^c^Microbial Interactions and Microbiome Center, University of Washington, Seattle, WA 98109

**Keywords:** Patescibacteria, epibiont, interbacterial

## Abstract

Patescibacteria, also known as the Candidate Phyla Radiation, are a diverse clade of largely uncultivated, small bacteria that comprise a significant proportion of all bacterial diversity. The few members that have been cultivated exhibit a fascinating life cycle in which they grow as obligate epibionts on the surface of host bacteria. In this Perspective, we make the case that the study of these unique, divergent, and poorly characterized organisms represents an exciting frontier in microbiology. This burgeoning field has already achieved several critical breakthroughs, including metagenomic sequence-based reconstructions of the metabolic and biosynthetic capabilities of diverse Patescibacteria and the development of generalizable strategies for their cultivation and genetic manipulation. We argue these that advances, among others, should pave the way toward a molecular understanding of the complex interactions that undoubtedly underpin the relationship between Patescibacteria and their hosts.

## Significance

Patescibacteria, also known as the Candidate Phyla Radiation, are an understudied but fascinating group of diverse and largely uncultivated bacteria. These ultrasmall microbes have reduced genomes lacking many typically essential pathways and appear to compensate for this deficiency by growing on the surface of host bacteria. The molecular mechanisms by which they do so and how they regulate their unique cell cycle remain virtually unknown. With the advent of new tools for the cultivation and genetic manipulation of Patescibacteria, we argue the time is ripe for researchers to begin investigating the reservoir of unexplored biology they encompass.

The past few decades have been good to bacteriologists. Indeed, those working in the field today are in the midst of and perhaps participating in one or more new and thriving frontiers that are keeping the discipline growing and vibrant. Ushered in by the discovery of CRISPR and other advances, bacteriophage are, as they once were, part of the daily vernacular of microbiologists. Although the first time bacteriophage held the spotlight, they gave us insights into the underpinnings of cellular biology, this time they are providing the organismal conflict needed to unravel the origins of innate and adaptive immunity (1–3). Another palpable frontier centers around the concept of bacteria as members of complex assemblages. Although the recognition that bacteria grow among other microbes dates back to the origins of the field, an appreciation for the ubiquity and complexity of microbial communities, as well as their health and environmental implications, did not enter the mainstream until around the turn of this century (4). The technological output from research in these areas is staggering: therapeutic gene editing, pathogen biosensors, and the promise of designer bacterial consortia for treating human gastroenterological diseases to name a few (5–7).

The Patescibacteria are a phylum of monoderm bacteria that constitute a substantial proportion of all microbial diversity. If this name is unfamiliar, you may be aware of another designation these bacteria fall within, the Candidate Phyla Radiation (CPR) (Box 1). Patescibacteria are found in ground water, surface fresh water and marine habitats, soil, the rhizosphere, and in stable association with virtually every human being (8–21). They can account for over 10% of bacteria in such habitats and yet because of impediments to cultivation have remained mostly outside the reach of experimentalists (13, 22, 23). However, a group of human oral cavity-associated Patescibacteria were rescued from the depths of microbial dark matter when their obligate growth as epibionts on host Actinobacteria was discovered (24). More recently, the natural competence of this same group of Patescibacteria was revealed and exploited to develop tools for their genetic manipulation (25).

In this piece, we highlight early findings and interesting open questions related to Patescibacteria. Our aim is to offer the perspective that with tractable experimental systems now in place, the fascinating biology of this large swath of microbial diversity makes a compelling subject for another exciting frontier in microbiological research.

## What Are Patescibacteria?

**Fig. 1.**
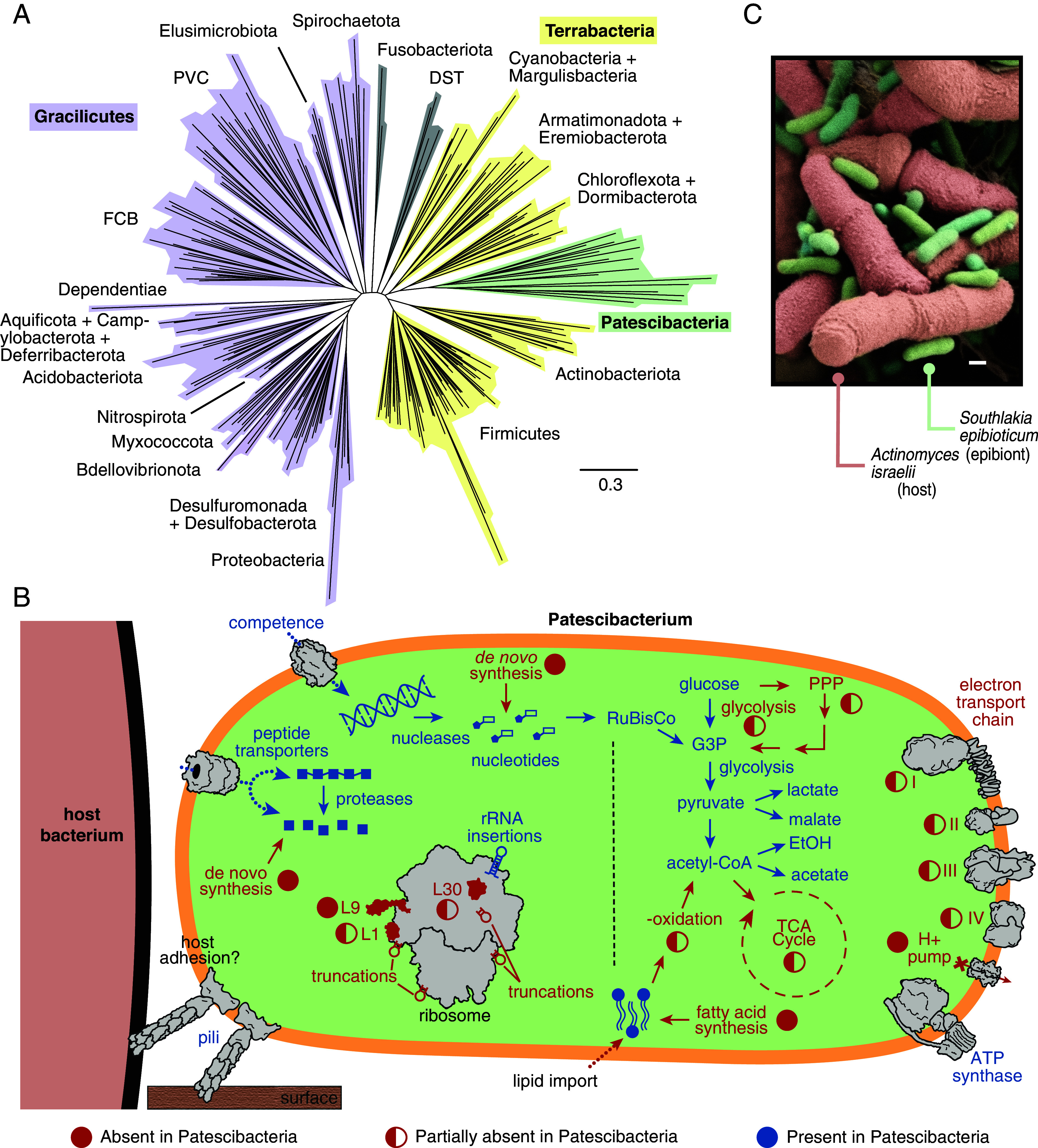
Patescibacteria are a diverse, monophyletic clade of bacteria with limited biosynthetic and catabolic capabilities. (*A*) Phylogeny indicating the relationship between Patescibacteria and other bacterial phyla, adapted from Coleman et al. and reprinted with permission from AAAS (69). The tree was generated using 62 concatenated marker genes, and the root was inferred using gene tree-species tree reconciliation. We have chosen this representation as several lines of evidence suggest it is more likely representative of evolutionary history than trees rooted using an archaeal outgroup, which suggest a more basal position for Patescibacteria (69). Patescibacteria are encompassed within Terrabacteria (monoderms) but are differentially colored for emphasis. FCB, Fibrobacterota, Chlorobiota, Bacteroidota; PVC, Planctomycetota, Verrucomicrobiota, Chlamydiota; DST, Deinococcota, Synergistota, Thermotogota. (*B*) Schematized patescibacterial cell indicating the presence or absence of key functions, as determined based on literature consensus. Filled red circles indicate pathways that are primarily or entirely absent from patescibacterial genomes; half-filled circles indicate pathways for which some components are common but others appear to be missing (i.e., glycolysis, the pentose phosphate pathway (PPP), the TCA cycle) or functions with variable distribution across the phylum; universally present structures are labeled with blue text. Unusual features of patescibacterial ribosomes are also indicated. (*C*) False-colored scanning electron micrograph obtained by our laboratory of the indicated Patescibacterium–host pair.

The Patescibacteria are a monophyletic group of ultrasmall (100 to 300 nm width) bacteria whose reduced genomes differ substantially in sequence and content from those of well-characterized organisms (Fig. 1*A*) (26, 27). Due to their small size and exceptionally divergent ribosomal gene sequences often containing introns, Patescibacteria were long undersampled in environmental sequencing studies (9, 28, 29). However, with the application of 16S rRNA primers designed to capture the variation in Patescibacteria sequences, expanded use of small pore size filters (<0.2 μM), and deeper sequencing, it is now apparent that Patescibacteria occupy a wide range of habitats (8–21).

The reduced genomes of Patescibacteria have exceedingly diminished catabolic and anabolic capacity (Fig. 1*B*). Catabolically, they lack key steps of glycolysis, the tricarboxylic acid cycle, and oxidative phosphorylation (26, 30, 31). Most Patescibacteria are thought to obtain energy via anaerobic fermentation of products released by the activity of the numerous glycoside hydrolases they encode, which are predicted to degrade a variety of complex carbohydrates (26, 32). Surprisingly, despite lacking a respiratory chain to generate the proton gradient needed to drive ATP synthesis, nearly all Patescibacteria encode an F-type ATP synthase (26). Hydrogenases encoded by some species could serve the role of the respiratory chain, but these proteins are not widely distributed across the phylum (31, 33). Alternatively, the ATP synthase may operate in reverse to drive antiporters or contribute to pH homeostasis (26, 34). Although evolutionary analyses suggest the lack of electron transport is an ancestral trait of Patescibacteria, some lineages appear to have horizontally acquired genes for anaerobic or even aerobic respiration, such as the complete sulfur oxidation pathway found in species colonizing a sulfide-rich spring, nitrate or nitrate reduction genes detected in multiple lineages, and a cytochrome *bo_3_* ubiquinol terminal oxidase operon found in the genomes of rhizosphere-colonizing species (17, 31, 35). In other species, partial pathways for sulfate or nitrate reduction are likely insufficient to support anaerobic respiration but may contribute to inorganic nutrient cycling (32, 33, 36, 37).

Among the most fundamental biosynthetic pathways missing from Patescibacteria are those for synthesizing amino acids, nucleic acids, and lipids (26, 38). Consistent with this, they encode an abundance of predicted energy-dependent solute uptake mechanisms and the competence machinery involved in DNA import (26, 38). In conjunction with an extensive array of proteases, DNA degrading enzymes, and nucleotide interconverting pathways, these may provide a source of amino acids and nucleotides (26). However, in the first genetic analysis of a Patescibacterium, competence machinery genes proved necessary for natural transformation but dispensable for growth, indicating this organism must encode an alternative means of nucleotide acquisition (25). Possible lipid uptake and breakdown mechanisms have not been identified in Patescibacteria, but an in situ analysis of subsurface aquifer samples suggests the source of these molecules may be lysolipids from neighboring bacteria (39).

Given their limited biosynthetic capacity, researchers long predicted that Patescibacteria live as symbionts or parasites, relying on a host to propagate (26). This prediction is supported by the discovery that multiple species of Patescibacteria from the Saccharibacteria class—to date, the only patescibacterial group that can be reliably propagated in vitro—grow as epibionts on the surface of Actinobacteria hosts (24, 40–44). Indeed, scanning electron micrographs of several Saccharibacteria species, including the first cultivated Saccharibacterium, *Nanosynbacter lyticus* TM7x, and the first to be genetically manipulated, *Southlakia epibionticum,* strikingly illustrate the intimate association between these organisms and their hosts (Fig. 1*C*) (45). Additional imaging studies provide evidence that patescibacterial species outside Saccharibacteria exhibit a similar growth mode, suggesting that epibiosis may be conserved across the phylum (46–48). This contrasts with other examples of epibiotic bacteria, such as *Bdellovibrio exovorus, B. qaytius,* and certain α-proteobacterial predators, in which the lifestyle appears to have evolved convergently from endo-parasitic or free-living organisms (49, 50). The relatively ancient origin of epibiosis among Patescibacteria (c.a. 3.7 billion years ago) is reflected in their streamlined genomes, which have lost considerably more biosynthetic capacity than other epibionts and encode systems apparently specialized to support host association (25, 27).

With the advent of cultivation techniques and tools for genetic manipulation for Saccharibacteria, researchers have begun to make headway toward experimental characterization of the cellular physiology of Patescibacteria. The first genome-wide survey of functions required for viability of a Saccharibacterial species revealed the essentiality of numerous functions typically dispensable for growth in other organisms (25). These include type IV pili (T4P), a predicted type IV secretion system (T4SS), the arginine deiminase pathway and multiple surface-associated proteins predicted to function as adhesins. The essentiality of T4P stems at least in part from their role in motility; time lapse microscopy studies reveal that the Saccharibacteria life cycle includes a motile phase, in which progeny released from attached, dividing cells rely on T4P extrusion to reach and establish an association with a new host cell (44). The role of the T4SS in Saccharibacteria is completely unknown, but researchers have speculated that it may deliver effectors to host cells (51). The arginine deiminase pathway provides a means of synthesizing ATP via conversion of arginine to ornithine and appears to promote survival of the unattached, nondividing cells (52). The distinctive and unusual biology revealed through these initial studies together with the many open questions remaining regarding these organisms serve to underscore the rich potential represented by a dedicated expansion of Patescibacteria research.

## Future Directions for the Field

Patescibacteria stand out in this era of microbiology for the breadth and fundamental nature of the many outstanding questions they present. These encompass everything from the molecular mechanisms by which they interact with host bacteria to the ecological roles they play across diverse habitats. It is therefore an exciting moment in the field, as the introduction of tools for in vitro cultivation and genetic manipulation of Patescibacteria makes tackling these questions now feasible.

### The Nature of the Patescibacteria Relationship with Their Hosts.

The limited biosynthetic capacity of Patescibacteria necessitates their acquisition of essential metabolic building blocks directly or indirectly from other organisms. Though examples remain limited to a handful of species, laboratory studies suggest that Patescibacteria propagation is a burden to the host bacterium (24, 46–48, 53). However, the specific host–Patescibacteria pairings, overall microbial diversity, nutrient landscape, and stresses encountered during these in vitro studies do not reflect those found physiologically. It thus remains to be determined to what extent environmental circumstances influence the nature of the relationship between Patescibacteria and their hosts. Indeed, in the case of the Saccharibacteria–Actinobacteria relationship, two recent studies have proposed mechanisms by which Saccharibacteria could benefit rather than harm their host in a natural setting: i) epibiont-produced ammonia could protect the host against acid stress in the mouth and ii) Saccharibacterial infection could repress phage receptor expression of its host (52, 54). Although these specific benefits of infection by a Saccharibacterium may not be sufficient to establish a mutualistic partnership, they highlight the need for more study of the nature of the relationships between Patescibacteria and their hosts. Creative in situ approaches and ecological modeling of population dynamics both stand to shed light on where the Patescibacteria–host relationship falls along the parasitism–commensalism–mutualism continuum (55).

### The Distribution of the Epibiotic Growth Mode.

In part separable from the nature of the interaction between Patescibacteria and their hosts is the degree to which Patescibacteria growth requires physical association with a host. Imaging studies employing fluorescent in situ hybridization or electron microscopy demonstrate that Patescibacteria from multiple classes attach to other bacteria in environmental settings (46–48). Consistent with these observations, Patescibacteria growth in the laboratory is documented strictly in association with a host cell (24, 41). Indeed, cells are motile and nondividing until intimate host cell engagement is achieved, whereupon they rapidly enlarge and generate progeny (25, 44). Furthermore, the widespread distribution in Patescibacteria of genes encoding a T4P—a structure implicated in epibiotic growth in Saccharibacteria—suggests that this mode of growth may be conserved beyond cultivated representatives (26, 44). While such data suggest that obligate epibiotic growth may be a common or even universal characteristic of Patescibacteria, this conclusion is by no means incontrovertibly established. Unattached patescibacterial cells are readily detected in nature, and Stepanauskas and colleagues found them no more likely to physically associate with other bacteria than those from other phyla in environmental samples (30). It remains to be determined whether free Patescibacteria cells represent a nonreplicative growth phase or propagation in the absence of host attachment. One patescibacterial lineage was detected in abundance within the cytoplasm of a paramecium, suggesting that other symbiotic or parasitic lifestyles may have emerged during diversification of the phylum (56).

### Mechanisms of Nutrient Acquisition.

For those Patescibacteria that unambiguously grow as obligate epibionts, it is presumed that they directly acquire essential nutrients from a host cell. Yet, we know very little about how this is achieved. Relative to their limited metabolic capacity, patescibacterial genomes are replete with genes encoding membrane and cell surface-associated proteins (27). These include lytic transglycosylases and phospholipases that have been proposed to compromise the host cell envelope, facilitating nutrient release (47, 48). They also include a large number of transporters, which appear capable of importing peptides, amino acids, and carbohydrates (26). Genes associated with DNA uptake through natural competence are among those enriched in Patescibacteria relative to other bacterial phyla, suggesting that this process may provide a source of nucleotides (27). However, the competence machinery in the Saccharibacterium *S. epibionticum* is dispensable for growth, suggesting at least some Patescibacteria obtain nucleotides through separate means (25). In Saccharibacteria, a candidate pathway to fulfill this role is the T4SS, which is conserved throughout the class and essential for growth in *S. epibionticum*. While these systems are more commonly involved in DNA or protein export, a T4SS from *Helicobacter pylori* is linked to DNA uptake, providing precedence for this function (57).

### Regulation of Differentiation.

To the extent that it has been characterized in Patescibacteria, epibiosis involves differentiation into at least two cellular states of disparate physiology. Sessile host-attached mother cells must extract molecular building blocks from their host in order to generate progeny, while unattached and nondividing swarmer cells are motile and presumably starved for many nutrients. The mechanisms governing differentiation between these states remain completely unknown. Given the dramatic proteome remodeling that undoubtedly underlies this process, translation regulation is likely to be key. Indeed, patescibacterial ribosomes and other proteins involved in translation regulation possess a number of unique features that may represent adaptations to support this transition. The rRNAs of Patescibacteria appear to be evolving more rapidly than their protein-coding genes and include sequence truncations and insertions not found in well-characterized bacteria (Fig. 1*B*) (9, 26, 58–60). Ribosomes in these organisms are also missing three proteins conserved among free-living bacteria. Finally, Tn-seq analysis of *S. epibionticum* revealed that several translation regulators typically dispensable for growth are required in this organism, including the ribosome hibernation protein RaiA, translation gating protein EttA, a ribosome release factor, and a tRNA methyltransferase (25). Determining how these features support the Patescibacteria life cycle and contribute to differentiation awaits study.

### Ecological Roles.

**Fig. 2.**
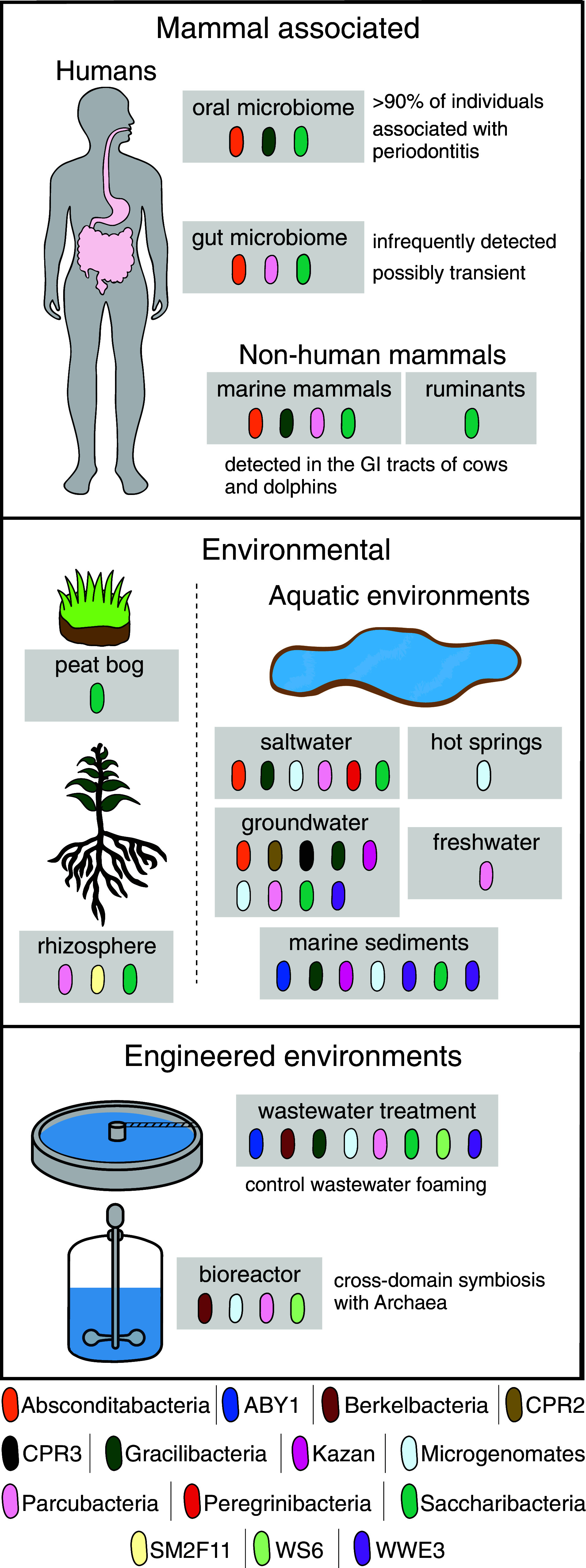
Environmental distribution of Patescibacteria. Specific classes of Patescibacteria detected in each habitat are depicted. Saltwater refers to both marine and hypersaline lake habitats. Distribution information obtained from both 16S rRNA gene surveys and metagenomic sequencing (both bulk and following single-cell separation). NCBI taxonomy was used to designate classes, and only those classes with >8 members are depicted. See main text for citations.

Given their widespread distribution and the fact that they must obtain critical nutrients from other bacteria, Patescibacteria are likely to play a significant role in microbial community ecology (Fig. 2). Assuming the relationship is widely parasitic, as preliminary observations suggest, Patescibacteria stand to have an impact akin to that of phage, which are believed to be important drivers of both bacterial community composition and bacterial evolution (61) The magnitude of the influence Patescibacteria exert in a given community will naturally depend on their abundance, which is a question that remains surprisingly unresolved. For example, when V4-targeting primers redesigned to capture known variability in patescibacterial 16S rRNA sequences were applied to global collection of wastewater treatment plant samples, the prevalence of patescibacterial sequences among amplicons increased from an average of 1.5% to 18.5% (13). Patescibacteria abundance has been characterized most systematically in groundwater, where they comprise a relatively high proportion of total bacteria (as much as 31%) (23). In this habitat, the presence of partial pathways for denitrification, sulfite reduction, and hydrogen production in patescibacterial genomes has prompted speculation that they play an important role in geochemical cycling (31, 33). However, none of these pathways have been studied experimentally, and it remains to be determined whether missing components are supplied by other organisms, or by divergent, difficult to identify proteins within Patescibacteria. Advancing our understanding of the ecological contributions of Patescibacteria will require deep metagenomic sequencing of diverse environmental samples that capture ultrasmall organisms.

### Impacts on Human Health.

A second environment in which Patescibacteria abundance has been more thoroughly characterized is the human microbiome. Several classes of Patescibacteria are detected sporadically in the gastrointestinal tract (16). An outstanding question is whether these organisms are established members of the gut microbiome or whether their presence is transitory, potentially stemming from groundwater consumption. In contrast, Saccharibacteria are clearly a common constituent of the human oral microbiome. Interestingly, observational studies have repeatedly found a correlation between Saccharibacteria abundance and periodontal disease in human oral clinical samples (16). Whether or not this is a causal relationship or the result of overall community changes during inflammation remains unresolved. In one study that sought to address this question, the experimental introduction of Saccharibacteria in a murine model of periodontitis was, on the contrary, associated with decreased inflammation and bone loss (62). The specific host Actinomycete strain employed in this study is linked to oral disease; therefore, the protective nature of coinfection with Saccharibacteria in this system is consistent with effects exerted on host bacteria. With the advent of genetic tools for Saccharibacteria, studies that characterize mechanisms by which Saccharibacteria influence oral disease are now within reach.

## Challenges Ahead in Patescibacteria Research

Despite the nearly three decades elapsed since the discovery of Patescibacteria, progress toward characterization of their physiology remains limited. Recent breakthroughs in the field underscore the unique biology that stands to be uncovered by a fresh infusion of ideas, funding, and tool development toward the study of these organisms. Realizing this potential will require overcoming a number of challenges, as we detail below.

### Challenges to Cultivation.

A major hurdle that must be overcome in order to expand mechanistic studies of Patescibacteria beyond Saccharibacteria is establishing methods for laboratory cultivation of additional lineages. Assuming most Patescibacteria require a host organism to support their growth, these efforts will necessarily require identification of the hosts for many more species. Two strategies show promise in this regard: Emulsion, Paired Isolation and Concatenation PCR, which detects physically associated cells collected from environmental samples, and a reverse genetics approach in which antibodies designed to target surface antigens of Patescibacteria strains of interest enable isolation of these cells and attached hosts using fluorescence activate cell sorting (42, 44). However, host organism identification is only a first bar that must be cleared toward establishing robust in vitro cultivation protocols. Several studies report examples of Patescibacteria–host enrichments that fail to achieve sufficient Patescibacteria levels to perform additional experiments or which cannot be stably maintained (42, 47, 48). Genomic analyses of target host-Patescibacteria pairs toward the design of optimal culture conditions may prove fruitful for overcoming this challenge. Another approach, which has been successful for Saccharibacteria, is to use readily culturable host strains related to those found associated with Patescibacteria in environmental settings as “bait” to enrich for compatible epibionts (41, 43). Broad application of these and potentially additional creative strategies will be critical for unlocking the rich biological diversity represented in the Patescibacteria phylum.

### Limitations of Current Genetic Tools for Patescibacteria.

Advancement in the study of patescibacterial cellular physiology would benefit tremendously from the expansion of the currently available limited genetic toolkit. Even fundamental techniques such as complementation, inducible expression, and transcriptional reporters have yet to be developed for Patescibacteria. Natural plasmids have yet to be discovered in these organisms, raising questions about the feasibility of employing traditional replicating plasmids or shuttle vectors as platforms for such genetic tools. However, if the natural transformation exhibited by Saccharibacteria is a widespread trait, as genomic analyses would suggest, this limitation could be overcome through the use of integrating cassettes, such as those as commonly employed in naturally transformable *Bacillus subtilis.* A particularly enticing potential use of the natural transformability of Patescibacteria lies in the development of CRISPRi technology. This method allows for the conditional knock-down of gene expression by targeted inhibition of transcription and can be used to perform genome-wide screens of conditional gene essentiality (63). It can also be used to study specific phenotypes linked to knock-down of essential genes, as discussed further below. The requirements to perform CRISPRi include a means to introduce sgRNAs targeting genes of interest and a system to control expression of a species compatible dCas9. Libraries of sgRNA expression constructs could be readily introduced to naturally competent Patescibacteria via natural transformation and targeted to integrate at a neutral genomic location via homologous recombination. A system for inducible expression in Patescibacteria, needed to control the timing of dCas9 expression as well as being useful in other contexts, requires development. Riboswitches represent promising candidates for gene expression modulation in Patescibacteria, as they function independently of the nature of the transcriptional machinery of the cell, merely requiring modification of the untranslated region of target transcripts and use of a cell-permeable ligand (64).

### Essentiality of Patescibacteria Genes.

A first Tn-seq study of a Patescibacterium revealed that, perhaps not surprisingly given their compact genomes, many of the genes required to sustain the unique aspects of the Patescibacteria lifestyle are essential for viability (25). Without the ability to make inactivating mutations, linking genes to specific phenotypes becomes challenging. Chemical biology techniques provide one means of overcoming this problem. A study by Du and colleagues in which they applied a chemical inhibitor of T4P extrusion to Saccharibacteria cultures illustrates both the potential utility and limits of such an approach: They demonstrate that T4P-mediated motility is required for the establishment of new host infections but could not evaluate whether T4P play a role in host attachment (44). Development of CRISPRi for Patescibacteria would provide a means to determine the impact of gene inactivation during specific growth phases. In the case of T4P, for example, the knock-down of minor pilins predicted to localize at the pilus tip at different points during the life cycle could be used to determine whether these proteins are required for motility, host attachment, or both. The development of nanobodies or synthetic peptide binders designed to target proteins of interest could also be a useful tool for linking the numerous essential genes with no characterized homologs to phenotypes, as a first step toward uncovering their functions (65).

### Limited Funding.

Given the widespread association between multiple Patescibacteria classes and the human microbiome and the likely parasitic nature of the interactions between these bacteria and their hosts, they stand to play an important role in shaping human-associated microbial communities and thus human health. However, moving beyond correlative abundance studies toward a mechanistic analysis of the impact of Patescibacteria and their interactions with their host bacteria on human health will require a significant funding commitment from the NIH. Funding initiatives from other agencies, such as NSF, DOE, and USDA, will be crucial for efforts to expand in vitro, mechanistic studies to encompass environmental Patescibacteria lineages beyond Saccharibacteria. These studies are key to unlocking the potential for the unique biology of Patescibacteria to yield new biotechnological tools and fundamental insights from this emerging frontier in microbiology.

**Box 1. Patescibacteria or CPR?**

There are two widely used names for the monophyletic group of bacteria that are the subject of this piece: Patescibacteria and Candidate Phyla Radiation (CPR). These overlapping monikers are the fallout of the nonlinear pathway leading to our present understanding of the phylogenic relationship between the constituent bacteria.

Sequences deriving from Patescibacteria were first detected in environmental 16S rRNA gene clones beginning in the mid 1990s. Researchers assigned the organisms various names; by convention, these reference the location of their discovery. For example, TM7 refers to the middle layer of a peat bog (torf mittlere schicht) in Germany, OP11 and OD1 (OP11-derived) to Obsidian Pool in Yellowstone National Park, USA, and GN02 to a hypersaline microbial mat in Guerrero Negro, Mexico (11, 12, 14, 21). Interest in these uncultivated candidate phyla received a boost in the early 2000s when researchers conducting bacterial diversity surveys of the human mouth identified TM7 bacteria in 96% of subgingival plaque samples across 46 individuals (22). Indeed, TM7 can constitute up to 2% of bacteria in these samples, enabling their microfluidic-based single-cell isolation and the first draft genome sequences of Patescibacteria (66). Nevertheless, with 16S rRNA gene or genome sequences of few representatives available, the evolutionary relationship between these and other candidate phyla was not apparent.

The threshold of sequenced representatives necessary to establish the relatedness among candidate phyla was reached in a landmark paper from Woyke and colleagues in 2013 (67). Their inclusion of 201 single-cell genomes, derived from largely uncultivated bacteria and archaea, into a rigorously constructed marker gene-based phylogeny revealed the monophyletic relationship between the candidate phyla GN02, OD1, and OP11, which the authors renamed Gracilibacteria, Parcubacteria and Microgenomates, respectively. They proposed grouping these into a superphylum named “Patescibacteria,” deriving from the latin patesco (bare), to reflect their shared limited metabolic capabilities. Approximately 2 y later, Banfield and colleagues published another landmark paper, reporting an additional 789 draft genomes from organisms belonging to a monophyletic lineage encompassing Patescibacteria and a number of previously unidentified or poorly characterized candidate phyla, including TM7. Using a 75% 16S rRNA identity phylum-level cutoff, they argued that many candidate phyla within this lineage should be subdivided into numerous additional phyla and referred to their totality as the “CPR” (9). With valid claims for the legitimacy of both names, researchers have since used Patescibacteria and CPR for the same group of bacteria interchangeably—much to the confusion of those outside of the field.

Inherent in the name CPR is the implication that the bacteria within it constitute multiple phyla. However, a systematic effort to normalize the diversity within bacterial taxonomic ranks argued that those classified as CPR should constitute a single phylum (60). This was determined using a phylogeny constructed from 120 ubiquitous single-copy marker genes. The explanation for the discrepancy between this analysis and prior ribosomal gene-based studies appears to be the anomalously high rate of evolution of patescibacterial translational machinery (discussed elsewhere in this article), leading to an overestimation of the diversity represented by these bacteria. In the interest of removing an unnecessary impediment to following or entering the field, we therefore advocate for consistent use of the term Patescibacteria (or Patescibacteriota, the phylum name to be adopted by the Genome Taxonomy Database on the basis of SeqCode recommendation (68) in future studies of these organisms.

## Data Availability

There are no data underlying this work.
